# Lymphome de Burkitt de l'enfant révélé par une invagination intestinale aigüe

**DOI:** 10.11604/pamj.2016.24.80.8500

**Published:** 2016-05-25

**Authors:** Mohammed El Fahssi, Khalid Sair

**Affiliations:** 1 Service de Chirurgie Viscérale, Hôpital Militaire d'Instruction Mohammed V, Rabat, Maroc

**Keywords:** Invagination intestinale, lymphome de Burkitt, tuméfaction mandibulaire, Burkitt lymphoma, intussusception, childhood

## Image en médecine

C'est un garçon de 13 ans qui a présente dans les suites d'une extraction dentaire une tuméfaction mandibulaire droite avec hypertrophie gingivale en regard (A). Trois jours plus tard apparition d'un syndrome occlusif franc avec à l'examen abdominal une masse sensible du flanc droit. Un scanner abdominal réalisé conclu à une occlusion grêlique en montrant une image en cocard compatible avec le diagnostic d'invagination intestinale. Opéré après une courte réanimation, l'exploration trouve une invagination grêlo-grêlique sur tumeur à un mètre vingt de l'angle de treitz sans nodules intra-péritonéaux, ou hépatiques et sans adénopathies mésentériques. Le geste opératoire a consisté en une résection segmentaire d'environ 30 cm du grêle L'ouverture de la pièce opératoire trouve plusieurs tuméfactions en médaillons de taille variable à bord surélevé (B) qui prennent naissance sur le versant mésentérique du grêle et visualise la grosse tuméfaction de 4 cm de grand axe (flèche) sur laquelle l'invagination grêlique s'est produite. L'examen anatomopathologique de la pièce opératoire montre qu'il s'agit d'une localisation grêlique d'un lymphome malin non hodgkinien à cellules B type lymphome de burkitt. Une chimiothérapie a aussitôt été démarrée avec rémission complète spectaculaire. Deux messages se dégagent de notre cas clinique le premier pour le dentiste en particulier qui devant tout tableau d'abcès dentaire inhabituel chez l'enfant il faut évoquer le diagnostic de lymphome de burkitt et le deuxième pour le clinicien qui devant toute invagination intestinale de l'enfant, l'origine lymphomateuse doit être évoquée à fortiori un lymphome de burkitt qui reste une urgence diagnostique et thérapeutique.

**Figure 1:**
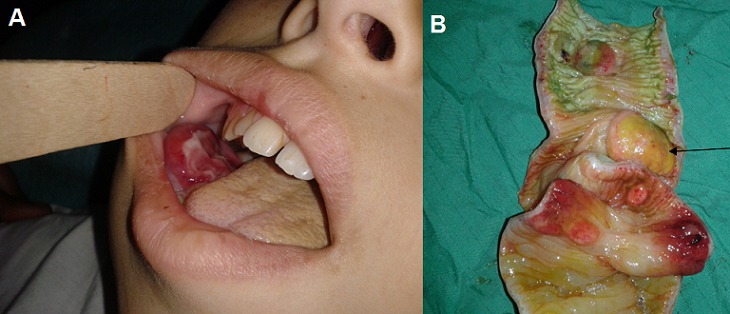
A) tuméfaction mandibulaire droite et hypertrophie gingivale en regard; B) tuméfactions en médaillon de taille variable à bord surélevé dont la plus grande (flèche) est la source de l'invagination

